# Acute Respiratory Distress Syndrome in Lemierre's Syndrome

**DOI:** 10.1155/2014/712946

**Published:** 2014-07-20

**Authors:** Paul N. Hein, Maida V. Soghikian, Munveer S. Bhangoo

**Affiliations:** ^1^Department of Internal Medicine, Scripps Green Hospital, 10666 N Torrey Pines Road (403C), La Jolla, CA 92037, USA; ^2^Division of Chest and Critical Care Medicine, Scripps Green Hospital, La Jolla, CA 92037, USA

## Abstract

Lemierre's syndrome is an infectious disease defined by the presence of septic thrombophlebitis with associated embolic phenomenon, most commonly to the lungs. Here we present two cases from a single institution of acute respiratory distress syndrome (ARDS) developing as a result of Lemierre's syndrome in previously healthy young adult men. ARDS can occur as a consequence of pulmonary septic emboli and sepsis, both of which are well-described consequences of Lemierre's syndrome. We describe important diagnostic and management considerations in the care of patients with hypoxemic respiratory failure and Lemierre's syndrome. Essential components of management include prompt antibiotic therapy, lung-protective ventilation strategies, and supportive care.

## 1. Introduction

Lemierre's syndrome is a potentially life-threatening diagnosis characterized by septic thrombophlebitis of the internal jugular vein following an oropharyngeal infection. The most common cause is* Fusobacterium necrophorum*, an anaerobic Gram-negative bacillus species, although other causative organisms have been implicated [1]. Multiorgan system dysfunction occurs as a result of septic emboli. Although relatively uncommon, this condition appears to be rising in prevalence, possibly due to restrictive antibiotic prescribing patterns in primary care settings in patients with upper respiratory infections [2].

This diagnosis should be suspected with the characteristic appearance of multifocal pulmonary infiltrates on chest radiograph. Here we report our experience from a single center of two cases of Lemierre's syndrome between 2010 and 2013. Our patients were previously healthy men who developed ARDS. They required admission to the intensive care unit and respiratory support with mechanical ventilation. These cases highlight the potentially catastrophic consequences of this disease and important management considerations in a critical care setting.

## 2. Cases

### 2.1. Case 1

**Figure 1:**
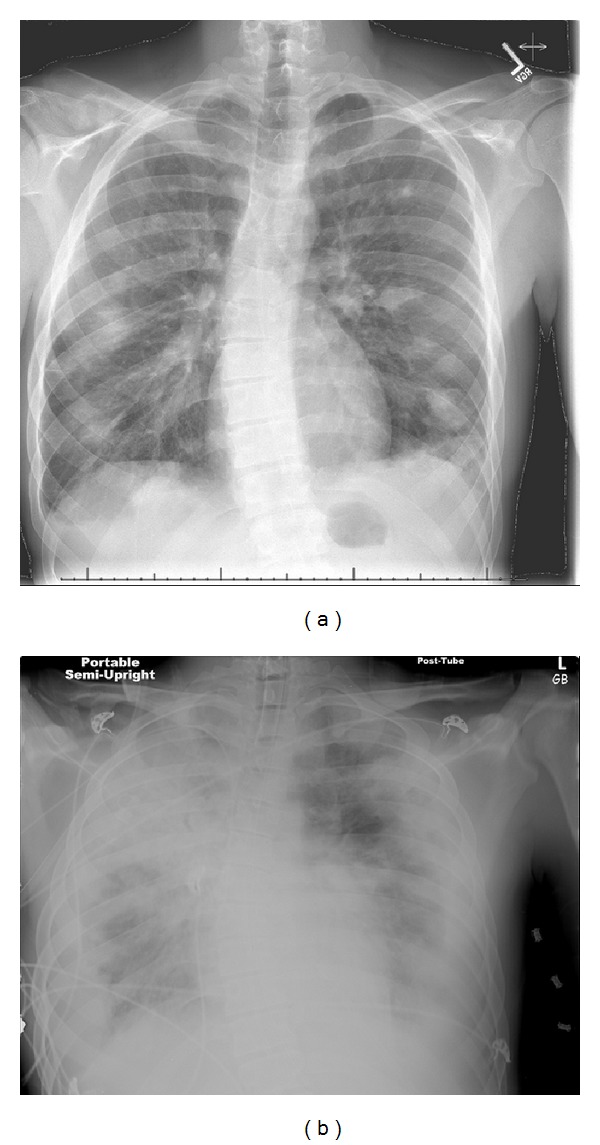
(a) Case 1 CXR early pulmonary nodules and infiltrates (cavitating on CT). (b) Case 1 CXR developed into dense bilateral infiltrates and ARDS.

A previously healthy 23-year-old man presented with ten days of sore throat, myalgias, and night sweats. On admission, the patient was febrile and tachycardic. Physical examination revealed posterior oropharyngeal erythema and shotty cervical lymphadenopathy. Chest radiograph revealed multiple patchy diffuse infiltrates (Figure 1(a)). Ultrasound of the neck showed nonocclusive thrombus of the left internal jugular vein extending into the proximal subclavian vein. Given these findings, the diagnosis of Lemierre's Syndrome was suspected. Computed tomography (CT) of the chest confirmed the presence of patchy bilateral cavitary lung nodules and pleural effusions.

Blood cultures later were positive for* Fusobacterium necrophorum* and confirmed the diagnosis. The patient's respiratory status rapidly decompensated five days into the patient's hospital course despite supportive care and prompt initiation of antimicrobial therapy. Repeat chest radiograph demonstrated worsening bilateral pulmonary infiltrates and pleural effusions (Figure 1(b)). Radiographic evidence, PaO_2_/FiO_2_ ratio of 88 mmHg, and the timeline of decompensation were consistent with diagnosis of severe ARDS. The patient was intubated and remained on mechanical ventilation for eight days after a seven-day weaning period (Table 1). The highest positive end-expiratory pressure (PEEP) and FiO_2_ required to maintain oxygenation were 5 cmH_2_O and 40% respectively, and albuterol/ipratropium bromide was given throughout ventilation. In addition, the patient required chest tube drainage of bilateral empyemas. The patient was started on unfractioned heparin to treat the internal jugular vein thrombus. However, he later developed an acute anemia requiring premature discontinuation of anticoagulation.

The patient's hospital course was further complicated by the development of pyomyositis of the right deltoid requiring surgical debridement and drainage. Abdominal imaging revealed a splenic infarct and transthoracic echocardiogram demonstrated a tricuspid valve vegetation consistent with endocarditis. The patient was discharged 23 days after admission and completed four weeks of outpatient treatment with metronidazole and aztreonam.

### 2.2. Case 2

A 23-year-old man presented to his primary care physician with complaints of four days of fevers, chills, and sore throat. The patient was sent home on azithromycin for suspected bacterial pharyngitis. The patient presented to the emergency room three days later with worsening symptoms. Physical examination revealed fullness and exquisite tenderness to palpation along the left neck as well as tonsillar exudates.

Laboratory evaluation was notable for leukocytosis with bandemia, acute renal failure, and elevated liver enzymes. CT of the chest demonstrated multiple peripheral nodules suspicious for septic emboli and bilateral pleural effusions. CT of the neck demonstrated thrombus and suppurative phlebitis of the left internal jugular vein. The diagnosis of Lemierre's syndrome was confirmed when blood cultures grew out* Fusobacterium necrophorum*. The patient was started on broad-spectrum antibiotic therapy and initiated on unfractioned heparin for treatment of the internal jugular vein thrombus.

The patient developed acute hypoxemic respiratory failure and septic shock requiring vasopressor support three days into his hospitalization. He was emergently intubated on a PEEP of 10 cmH_2_O and an FiO_2_ of 50% and was given albuterol and ipratropium bromide throughout intubation (Table 1). PaO_2_/FiO_2_ ratio of 89 mmHg, radiographic evidence of bilateral pulmonary infiltrates, and chronological disease progression were consistent with diagnosis of severe ARDS. Repeat CT scan of the chest showed heterogeneous fluid collections of the thorax requiring decortications and chest tube placement. Laboratory evaluation of the pleural fluid was consistent with empyema and hemothorax, at which point anticoagulation therapy was discontinued. The patient required blood product transfusion for acute blood loss anemia. The patient's respiratory status eventually improved and after a weaning period of five days he was extubated fourteen days later.

Twenty-eight days after presentation, the patient was discharged. He completed four weeks of antibiotic therapy of piperacillin/tazobactam and metronidazole. Interestingly, the patient represented to the hospital two years later with streptococcal pharyngitis. Repeat ultrasound of the neck demonstrated stable, chronic DVT in left internal jugular vein. Given that there was no extension of the original thrombus, the patient was discharged home without further anticoagulation.

## 3. Discussion

We present two cases from a single-center of previously healthy young men with illnesses that progressed rapidly from nonspecific upper respiratory symptoms to severe respiratory failure. In each of these cases, the findings of cavitary lung lesions on radiographic imaging heightened suspicion for embolic phenomena. The presence of internal jugular thrombosis (reported in 59% of cases) further supported the diagnosis of Lemierre's Syndrome. Although no uniform criteria exist for this condition, important components in the diagnosis include (i) a prodromal oropharyngeal illness, (ii) internal jugular vein thrombophlebitis, (iii) evidence of embolic phenomenon, and (iv) isolation of* Fusobacterium* species [3].

The lungs are the most frequently reported sites of metastasis in Lemierre's syndrome, occurring in 80–90% of patients [3–5]. Multiorgan involvement in this condition is common and occurs as a result of septic emboli. Other reported clinical manifestations include septic arthritis, renal failure, transaminitis, meningitis, abscess formation, and disseminated intravascular coagulation [6].

ARDS is a syndrome defined by hypoxemic respiratory failure associated with noncardiogenic pulmonary edema. The pathogenesis is related to diffuse alveolar damage precipitated by a proinflammatory state [7]. ARDS is most commonly associated with sepsis, most directly due to alveolar inflammation but septic shock-related injury may also be an additional contributory factor in severe cases [8]. The diagnosis requires a PaO_2_/FiO_2_ ratio of <300 mmHg with severe disease defined by values below 100 mmHg [9]. Other necessary components include noncardiac pulmonary edema with radiologic evidence of bilateral pulmonary infiltrates.

Based on these criteria, both patients in this series met criteria for severe ARDS. Because patients with Lemierre's syndrome may present to providers with nonspecific, mild respiratory symptoms, the diagnosis may not be considered until an advanced stage of the disease course. Both patients' respiratory status decompensated approximately seven to ten days after the onset of symptoms. This is consistent with one reported case in which ARDS developed eleven days after the onset of symptoms [10]. While ARDS occurring in Lemierre's syndrome has been described in case reports, its incidence in this condition is unknown [10–12]. The potential combination of severe sepsis and pulmonary emboli puts patients with Lemierre's syndrome at high risk of lung injury and hypoxemic respiratory failure. Treatment necessitates a strategy of lung protective ventilation, aggressive antibiotic therapy for treatment of sepsis, and supportive care [13]. While our patients required prolonged courses of mechanical ventilation, their FiO_2_ and PEEP requirements to maintain oxygenation were relatively modest. Interestingly, survivors of ARDS associated with Lemierre's syndrome appear to be free of significant long-term pulmonary sequelae, as was the case with our two patients [11].

Prompt initiation of effective antibiotic therapy is likely the most critical component in managing patients with Lemierre's syndrome. Antibiotics should be tailored against* Fusobacterium* species. Metronidazole is an appropriate first-line antibiotic for this condition, although resistant strains are reported [14]. In both cases, delayed recognition of the condition may account for the development of overwhelming sepsis and severe respiratory failure. Ultimately, septic thrombophlebitis in a young person should raise suspicion for Lemierre's syndrome and should warrant an aggressive search for systemic complications including ARDS [15].

## 4. Conclusion

Lemierre's syndrome is a systemic illness that can progress rapidly from a nonspecific pharyngitis to acute respiratory failure. While direct lung injury from septic emboli is well described, patients also are at risk for developing ARDS. Medical providers in a critical care setting should have a high suspicion for the development of ARDS in a patient with Lemierre's syndrome.

## Figures and Tables

**Table 1:** Summary of relevant case characteristics.

	Case 1	Case 2
Patient characteristics		
Age (years)	23	23
Sex	Male	Male
Duration of stay (hospital/ICU)	23 days/20 days ICU	28 days/23 days ICU
Causative organism	*F. necrophorum *	*F. necrophorum *
Disease characteristics		
Multifocal cavitary pneumonia	Yes	Yes
Mechanical ventilation (duration)	Intubated, 8 days	Intubated, 14 days
Pleural effusions/thoracostomy	Loculated pleural effusion, bilateral thoracostomy	Pleural effusion/hemothorax, bilateral thoracostomy
Renal failure	Yes	Yes
Liver dysfunction	Yes	Yes
Extrapulmonary manifestations	Pyomyositis, splenic infarction, tricuspid valve endocarditis	Peritonsillar abscess
Labs, peak values		
WBC count (k)	20.9	42.4
Hemoglobin/HCT (g/dL)	7.4	6.6
Platelets (trough/peak, k/uL)	60/618	18/1070
INR	2.3	2.9
GFR (mL/min)	28	28
Ventilation		
Max PEEP (cmH_2_O)	5	10
Max FiO_2_ (%)	40	50
Patient positioning	Fowler's/Semi-Fowler's	Fowler's/Semi-Fowler's
Inhaled therapies	Albuterol/ipratropium bromide	Albuterol/ipratropium bromide
Weaning time (days)	7	5
Interventions		
Antibiotics	Metronidazole/aztreonam	Metronidazole/Zosyn
Anticoagulation	Heparin (d/c anemia)	Heparin (d/c hemothorax), warfarin on discharge
Blood products	Transfused PRBC's	Transfused PRBC's
Vasopressor	—	Yes
Surgical interventions	Right deltoid abscess drainage/debridement	Unsuccessful peritonsillar abscess drainage
